# The complete chloroplast genome sequence of Sainfoin (*Onobrychis viciifolia*)

**DOI:** 10.1080/23802359.2020.1871439

**Published:** 2021-02-11

**Authors:** Zhongzhiyue Jin, Wenbo Jiang, Dengxia Yi, Yongzhen Pang

**Affiliations:** Institute of Animal Sciences, Chinese Academy of Agricultural Sciences, Beijing, China

**Keywords:** Chloroplast genome, Sainfoin, Fabaceae, *Onobrychis viciifolia*

## Abstract

Sainfoin (*Onobrychis viciifolia*) is one of the dominant legume forages distributed in Northern China. In our study, we assembled and annotated the structure of the complete chloroplast genome of sainfoin. The length of the circular genome is 122,102 bp. It contains 115 genes, including 79 protein-coding genes (68.7%), 31 tRNA genes (26.96%) and 5 rRNA genes (4.35%). The GC content of the total chloroplast genome of sainfoin is 34.58%. We construct the phylogenetic relationships between the chloroplast genome of sainfoin and the other 16 species by the Maximum likelihood (ML), and found sainfoin is most closely related to *Hedysarum petrovii* and *Hedysarum taipeicum*.

Sainfoin is a perennial herb of the *Onobrychis* genera, and the plants of this genera is distributed throughout temperate and subtropical regions of Eurasia, N and NE Africa (Amirahmadi et al. 2016). Sainfoin was recorded in North Africa. It is native to south Asia and has been cultivated for many years in Russia, Europe and many parts of Asia. Sainfoin was introduced into North America in 1786 (Bhattarai et al. 2016). As an excellent forage grass, sainfoin is widely cultivated in 23 provinces/autonomous regions of China, such as Gansu, Xinjiang, Inner Mongolia. Sainfoin is rich in protein and it also has high levels of condensed tannin (Jonker and Yu 2017). Ruminant animals fed with sainfoin that is rich in condensed tannins will not suffer from bloat (Lagrange and Villalba 2019). Meanwhile, moderate levels of condensed tannins in sainfoin have positive effects on digestion and absorption of amino acids, urinary nitrogen secretion and animal performance (Hayot Carbonero et al. 2011; Huyen *et al*. 2016; Jonker and Yu 2017). In addition, allelochemicals of sainfoin can inhibit the growth of weeds (Cheng and Cheng 2015).

Sainfoin can be grown in salty and arid regions, and chloroplast is one of the primary organelles affected by salt and drought stress (Beyaz et al. 2018; Beyaz 2019a, 2019b; Beyaz and Yildiz 2020). The chloroplast is an important organelle that has unique genome sequence, and the chloroplast genome of plants has been a hot spot in plant molecular evolution and systematics (Clegg et al. 1994). In this study, we report the complete chloroplast sequence and structure of sainfoin and analyze the relationship between sainfoin and other legume species by phylogenetic analyses.

Seeds of sainfoin was stored at the Forage Germplasm Bank at Institute of Animal Science of the Chinese Academy of Agricultural Sciences (Beijing, E116°29′, N40°03′). Plants of sainfoin was cultivated at Lang Fang City, Hebei province, China (E116°41′, N39°31′) in 2019, and young leaves were collected as molecular material. The voucher specimen (FR003) was deposited at the Herbarium of the Department of Grassland at IAS-CAAS, Beijing, China. Total genomic DNA was extracted from the fresh leaves of sainfoin with a DNA Extraction Kit from Tiangen Bio Tech Co., Ltd (Beijing, China). Using the Illumina Novaseq PE150 platform (Illumina Inc, San Diego), the DNA sample was sequenced. The software GetOrganelle v1.5 (Jin et al. 2018) was used to assemble the adaptor-free reads into a complete chloroplast genome, with the chloroplast genome of *Medicago truncatula* (GenBank accession number: NC-003119) as a reference. The complete chloroplast genome of sainfoin was annotated by the online program CPGAVAS2 (Shi et al. 2019) and GeSeq (Tillich et al. 2017), and then submitted to GenBank with the accession number MT528597.

The complete chloroplast genome of sainfoin is a typical circular structure with 12,2102 bp in size. The GC content of the total chloroplast genome of sainfoin is 34.58%. The chloroplast genome has 115 genes in total, containing 79 protein-coding genes, 31 tRNA genes and five rRNA genes. Among them, 28 genes are identified to encode amino acid transfer protein (*trnL-UAG*, *trnN-GUU*, *trnR-ACG*, *trnA-UGC*, *trnE-UUC*, *trnV-GAC*, *trnM-CAU*, *trnL-CAA*, *trnP-UGG*, *trnW-CCA*, *trnQ-UUG*, *trnS-GCU*, *trnR-UCU*, *trnC-GCA*, *trnD-GUC*, *trnY-GUA*, *trnE-UUC*, *trnT-GGU*, *trnS-UGA*, *trnG-GCC*, *trnM-CAU*, *trnS-GGA*, *trnT-UGU*, *trnL-UAA*, *trnF-GAA*, *trnM-CAU*, *trnK-UUU*, *trnH-GUG)*, 21 genes are identified to encode ribosomal structural proteins (*rpl32*, *rps15*, *rps12*, *rps7*, *rpl23*, *rpl2*, *rps19*, *rpl22*, *rps3*, *rpl16*, *rpl14*, *rps8*, *rpl36*, *rps11*, *rps12-fragment*, *rpl20*, *rps18*, *rpl33*, *rps2*, *rps14*, *rps4*), four genes are identified to encode ribosomal structural RNAs (*rrn5*, *rrn4.5*, *rrn23*, *rrn16*), 15 genes are identified to encode electron transport proteins (*ndhF*, *ndhD*, *ndhG*, *ndhI*, *ndhA*, *ndhH*, *ndhB*, *petD*, *petB*, *petG*, *petA*, *petN*, *ndhJ*, *ndhK*, *ndhC*), five genes are identified to encode light collection structural protein of photosynthesis (PSI) (*psaC*, *psaJ*, *psaI*, *psaB*, *psaA*), and 14 genes are identified to encode light collection structural proteins of photosynthesis (PSII) (*psbH*, *psbT*, *psbB*, *psbE*, *psbF*, *psbL*, *psbJ*, *psbK*, *psbI*, *psbM*, *psbD*, *psbC*, *psbZ*, *psbA*).

In order to further determine the phylogenetic position of sainfoin, we selected another 16 chloroplast genome sequences of Fabaceae deposited at the NCBI GenBank. The sequences were aligned by using MAFFT v7 (Katoh et al. 2019). In addition, the phylogenetic tree based on the common protein-coding genes of 17 species was then constructed by using raxmlGUI1.5b (v8.2.10) (Silvestro and Michalak 2012) via maximum likelihood (ML) method. It is clear that sainfoin is closely related to *Hedysarum petrovii* and *Hedysarum taipeicum* in the phylogenetic tree (Figure 1).Our present study will provide important sequence information for species identification, and phylogenetic relationship comparison in Fabaceae family, especially for legume forage.

**Figure 1.**
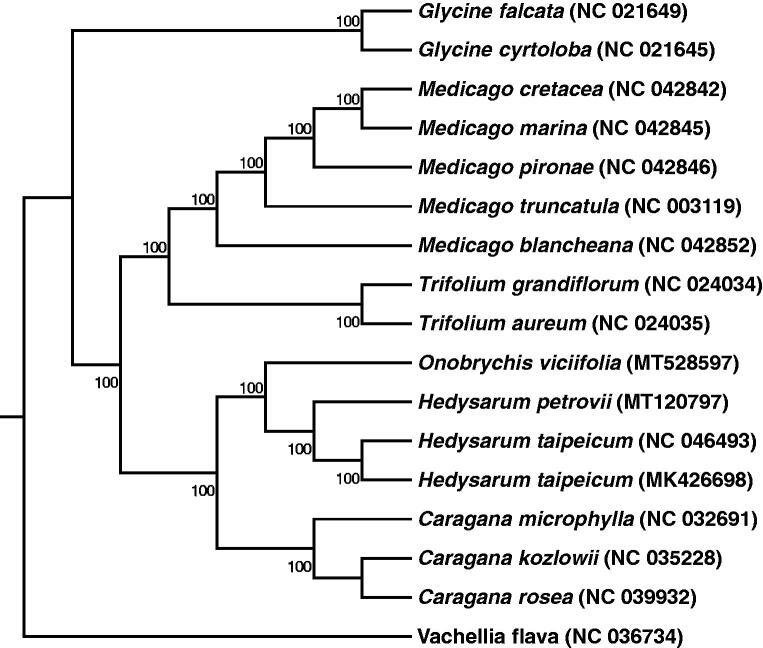
Phylogenetic tree reconstruction using maximum likelihood (ML) method based on a total of 17 complete chloroplast genome sequences of 17 species. Numbers above below the branch lines represent ML bootstrap values..

## Data Availability

The data that support the findings of this study are openly available in NCBI at Genbank with accession number MT528597 (https://www.ncbi.nlm.nih.gov/nuccore/MT528597). Raw sequencing reads used in this study was deposited in the public repository SRA with accession number SRR12763671 (https://www.ncbi.nlm.nih.gov/sra/?term=SRR12763671).
